# The Ultrasonic Investigation of Phase Transition in Olive Oil up to 0.7 GPa

**DOI:** 10.1007/s11746-013-2223-2

**Published:** 2013-03-12

**Authors:** A. J. Rostocki, R. Tarakowski, P. Kiełczyński, M. Szalewski, A. Balcerzak, S. Ptasznik

**Affiliations:** 1 Faculty of Physics, Warsaw University of Technology, ul. Koszykowa 75, 00-662 Warsaw, Poland; 2 Institute of Fundamental Technological Research, Polish Academy of Sciences, ul. Pawińskiego 5B, 02-106 Warsaw, Poland; 3 Department of Meat and Fat Technology, Institute of Agricultural and Food Biotechnology, ul. Jubilerska 4, 04-190 Warsaw, Poland

**Keywords:** Vegetable oils, Sound velocity, Triacylglycerols, Phase transition, High pressure

## Abstract

This paper presents measurements of sound velocity and attenuation in olive oil, with known chemical composition, as a function of pressure, within the range of pressure up to 0.7 GPa. Dependencies of sound velocity, relative ultrasonic wave attenuation, volume, and adiabatic compressibility on pressure show discontinuities. This proves the existence of the first order phase transition in olive oil (liquid to solid-like phase transition). Rapid and large changes in relative attenuation testify to the existence of a phase transition in olive oil. Moreover, the kinetics of phase transition was also investigated. Measurement of acoustic wave velocity and relative attenuation in olive oil during the phase transition and in the high-pressure phase is a novelty. The results obtained can be useful in the development of new methods in food (edible oils) control, processing, and preservation.

## Introduction

Vegetable oils are an important component of human diet because they provide nutritional properties, flavor, and consistency. Knowledge of their physical properties is indispensable. Recently intensive research on the pressure changes of physical properties of vegetable oils has been done in several laboratories. During food manufacture, processing and conservation, high-pressure methods are often used. For example, olive and seed oils are subjected to a pressure of 700 MPa [1]. Phase transitions that occur during the pressurization of oils have a very significant effect. Phase transformations are accompanied by drastic changes in the physicochemical and mechanical properties of oils [2]. In the case of oils such as castor oil, rapeseed oil, soy oil etc., first order phase transitions have been observed [3–5]. Since the common component of those materials is triacylglycerols of fatty acids (mostly of oleic acid), some comparative studies on pure triacylglycerols (laboratory synthetic) such as triolein have been conducted [6, 7]. They have shown the occurrence of the same type of phase transition as that in natural oils. First order phase transition was also observed in diacylglycerol (DAG) oil [8]. Further studies indicated a crystallization process during the phase transition and also observation of molecular crystal forms has been reported [9]. Although the literature on oil crystallization under the influence of temperature at atmospheric pressure is very extensive, see, e.g., [10], publications concerning the phase transition occurring in the oils under pressure are not yet numerous e.g., [2, 11].

Olive oil is one of the most frequently used in everyday life edible oils. Olive oil has been a subject of intensive research, also with regard to the effect of pressure because of its importance to the food industry. Several physical properties such as: thermal conductivity and density [12], ultrasonic attenuation coefficient [13], ultrasonic velocity [14], PVT characteristic up to 150 MPa [15], volumetric properties [16] and thermal diffusivity [17]have been presented by various authors, but none of them have reported pressure-induced phase transition. Some suggestions appeared in the work [18], but the results obtained were ambiguous.

Having earlier experience in sound velocity measurements under high pressure and using the method developed by the authors in [19, 20], we decided to investigate the olive oil by the ultrasonic method. Ultrasonic methods have been successfully used for various oils properties investigations [21–24].

## Experimental Procedures

Olive oil is one of the most popular edible oils and is produced in several countries. Because of various brands of the product available on the market, for our experiments we selected olive oil produced in Spain with a high content of oleic acid triacylglycerols.

Olive oil sample was analyzed by means of the gas chromatography method using a Hewlett-Packard HP6890 device with a Flame Ionization Detector and a high-polar column BPX 70. The analysis was conducted following the AOCS Cd 11b-91 method. The analysis was performed according to the ISO 5508 and ISO 5509 norms. The composition of the esters of fatty acids in the sample obtained from gas chromatography, are presented in Table 1. There are five major fatty acids: the dominant C18:1 *cis*-oleic acid, C16:0 palmitic acid, C18:2 *cis*–*cis*-linoleic acid, C18:0 stearic acid and C16:1 palmitoleic acid. Other fatty acids are presented in small amounts below 1 %. The positions of particular chains in the triacylglycerol molecules were determined for frequently occurring fatty acids. Those results are presented in Table 2, where the *sn*-1,3 means the external position, and *sn*-2 internal position in the triacylglycerol molecule.

**Table 1:** Composition of investigated olive oil

Fatty acid	C14:0	C16:0	C16:1	C17:0	C17:1	C18:0	C18:1c	C18:2 ct/tc
% in olive	< 0.1	11.02	1.01	0.1	0.1	3.4	76.8	< 0.1
	C18:2 cc	C18:3 ccc	C20:0	C20:1	C20:2	C22:0	C24:0	C24:1
	5.9	0.7	0.4	0.3	<0.1	0.1	<0.1	<0.1

**Table 2:** Position of fatty acids in olive oil molecules

Fatty acid	*sn*-2 TAG	*sn*-1,3 TAG
C 16:0	5.0	95.0
C 16:1	8.6	91.4
C 18:0	21.0	79.0
C 18:1	86.1	13.9
C 18:2	49.7	50.3
C 18:3	58.5	41.5

This information could be important when trying to compare the values obtained with results from other experiments.

**Fig. 1:**
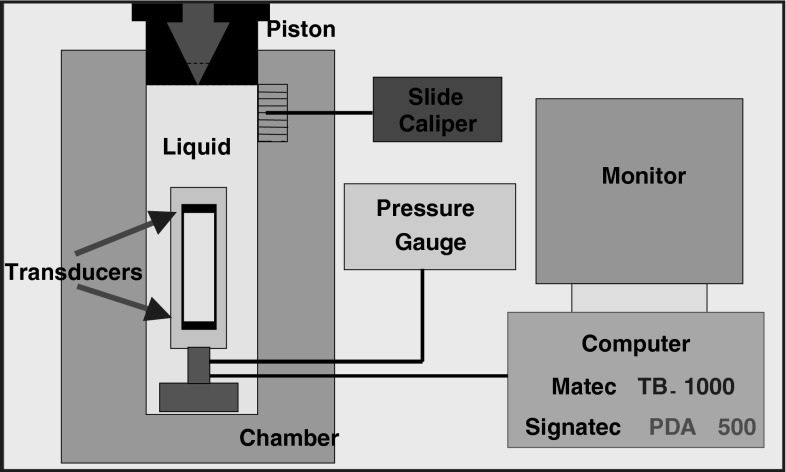
Experimental setup for measuring longitudinal ultrasonic wave velocity in liquids at high pressure

High-pressure measurements were performed using a setup designed and constructed by our team, see Fig. 1. Pressure was applied by a manually driven hydraulic press. All detectors were located along with the tested olive sample inside the 22-cm^3^ capacity pressure chamber. The pressure inside the chamber was measured by a Manganin sensor, calibrated using a dead weight piston gauge with a relative accuracy of 0.05 %. The standard deviation of the measurement series of resistance computed for 50 values of resistance was smaller than 1 mΩ [25]. Change in manganin sensor resistance in the range of pressures used in our measurements was approximately 1400 mΩ. Volume changes were measured by observation of piston displacement within the chamber. It was measured by a digital calliper gauge. Corrections related to the expansion of the chamber were evaluated from the Lamee equations and considered during data analysis. Therefore, the main source of errors in determining the volume was the error in measurement of piston displacement which was measured using a digital caliper with an accuracy of 0.01 mm. This gives a measurement error of the volume of the order of 0.1 %. A constant temperature of 293 K was maintained by a thermostatic water bath.

Initial density and specific volume of the sample were determined by weighing on an electronic weighing machine (± 1 mg) the known volume of olive oil. Since the mass of the sample did not change during the experiment, the changes in volume could be used to determine changes in these parameters versus pressure.

The sound velocity was measured using the setup developed by the authors and presented in detail in [19] and [20]. The measurement was based on the evaluation of the time of flight between two piezoelectric transducers. LiNbO_3_ (Y36 cut) plates (Boston Piezo-Optics Inc., USA) were used as both the transmitter and the receiver (frequency *f* = 5 MHz). The ultrasonic transducers’ mounting has been designed to provide a low level of parasitic ultrasonic signals. The signal to the transmitter was supplied with a TB-1000 pulser-receiver computer card (Matec, USA). After passing through the sample of olive the signal was detected by the receiver and was processed by the PDA-1000 digitizer card (Signatec, USA). To increase the signal to noise ratio, each signal was averaged 1024 times.

The distance between the transducers and pulse transition time between them were used to calculate the speed of sound in the medium. A cross-correlation method was used to measure the time of flight.

Ultrasonic velocity measurement is difficult and uncertain when using classical methods. Instead, it is possible to use methods based on digital signal processing such as the cross-correlation function [26]. The cross-correlation function *h*(*t*), between two functions *f*(*t*) and *g*(*t*), is defined by Eq. 1

1 $$ h\left( t \right) = \int\limits_{ - \infty }^{\infty } {f\left( \tau \right)} \,g\left( {t + \tau } \right)d\tau $$

The first signal received (the function *f*(*t*)) corresponds to the ultrasound pulse that travels across the distance *L* between the transmitting and receiving transducers. Part of the ultrasonic energy of the first signal is reflected at the receiving transducer back to the transmitting transducer, which in turn reflects part of the incident energy back to the receiving transducer. As a result, the next impulse detected by the receiving transducer (function *g*(*t*)) will travel an extra distance 2*L* between the transducers. In total, this signal travels the distance 3*L*.

The correlation analysis yields a measure of the similarity between the two considered pulses *f*(*t*) and *g*(*t*) shifted in time. Because those two pulses have a similar shape but a different amplitude and delay, the cross-correlation function reaches a maximum for *t* equal to the evaluated time difference corresponding to the distance 2*L*. The time delay was measured with a nanosecond resolution.

The relative uncertainty for the ultrasonic velocity in liquid equals ± 0.3 % at a 95 % confidence level.

The pressure was applied in increments of 10 MPa. Each pressure increase was followed by a time interval which allowed the olive oil to reach thermodynamic equilibrium. Approaching a pressure of 450 MPa we noticed a tendency of the pressure to decrease at a fixed position of the piston. This indicated that the phase transition was beginning. Therefore, at a pressure of 450 MPa the compression was stopped and the piston in the high-pressure chamber was locked into position to allow the phase transformation to occur undisturbed. Subsequently, pressure was still raised to 600 MPa, and later it was gradually reduced.

## Results and Discussion

**Fig. 2:**
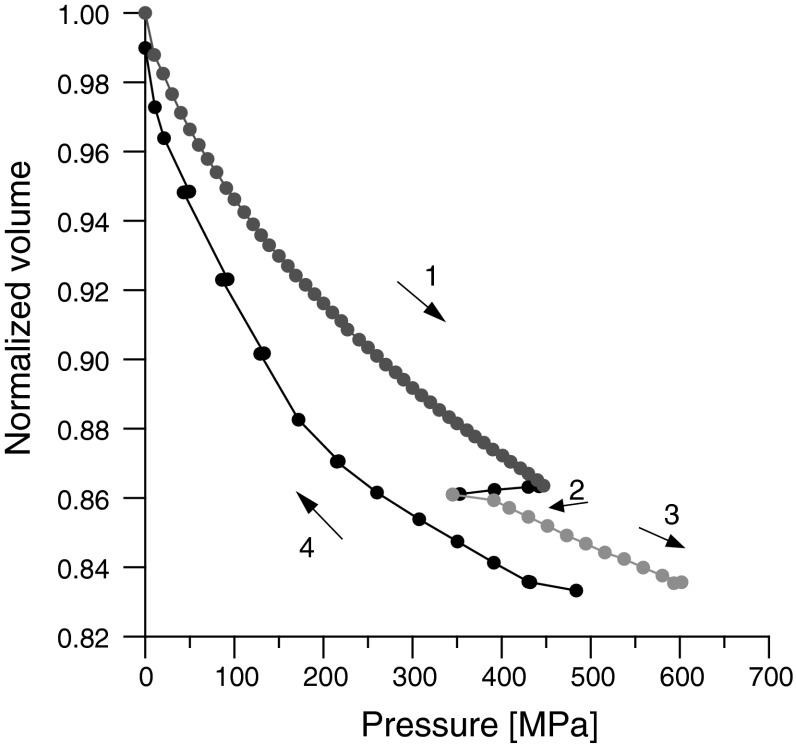
Relative volume changes in olive oil versus pressure, *T* = 293 K. *Arrows* indicate the direction of pressure changes. *1* low-pressure phase, *2* phase transition, *3* high-pressure phase, *4* decompression

During the experiment, the measurements of the volume dependence on pressure and sound velocity dependence on pressure were performed simultaneously. The changes of volume with pressure are shown in Fig. 2. As one can see at a pressure of about 450 MPa, the above-mentioned dependence has a discontinuity characteristic for the first order phase transition. For the pressure values below the phase transition, the data are in good agreement with those presented by Guignon [16]. Above the phase transition, the observed dependence of volume on pressure is very similar to that observed in triolein and described in detail in [7].

**Fig. 3:**
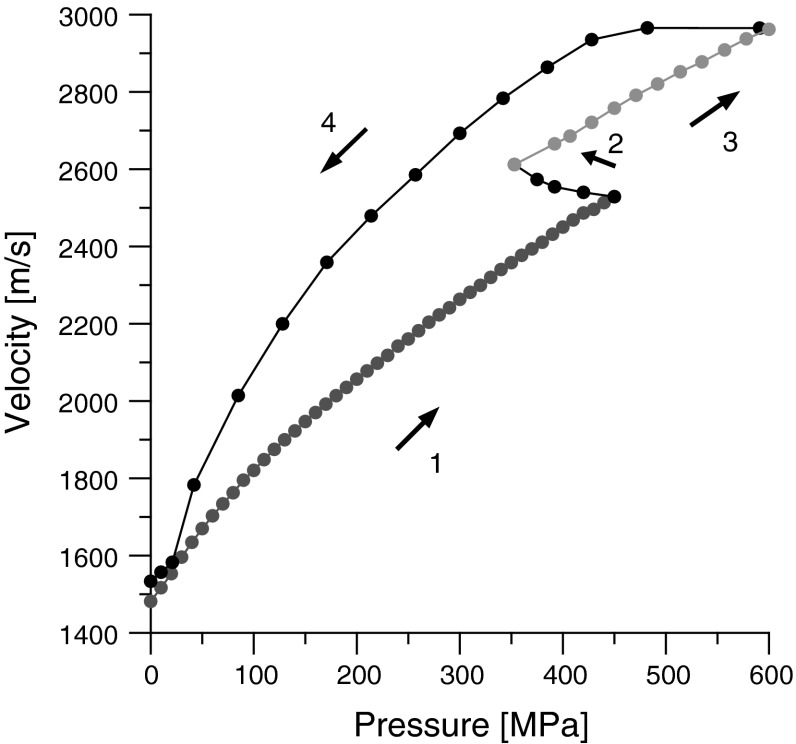
Speed of sound in olive oil as a function of pressure, *T* = 293 K, *f* = 5 MHz. *Arrows* indicate the direction of pressure changes. *1* low-pressure phase, *2* phase transition, *3* high-pressure phase, *4* decompression

The changes in sound velocity caused by the elevation of pressure are presented in Fig. 3. At a pressure relevant to the phase transition, the discontinuous increase in the speed of sound was noticed, despite the drop in pressure. The phase transition is defined as the transition of the first order if it is accompanied by an abrupt change in physical parameters, such as density [2]. We have observed such a phenomenon during the phase transformation in olive oil. The ultrasonic wave velocity depends among other things on the density, and therefore a change in density must produce a similar change in wave velocity. Observation of the measured wave velocity discontinuity as a method for detecting the phase transition has already been used in the work in [27]. Such phase transitions at similar pressures were present in all previously studied triglycerides [19]. At a pressure of 600 MPa, the speed of sound is almost double compared to its value at atmospheric pressure. Values of about 3000 m/s are typical for solid-like media, which confirms the crystallization of triglycerides observed in model triglycerides such as triolein and trilaurin, reported by Ferstl et al. in [9].

During the decompression, hysteresis of the sound velocity and volume changes were clearly visible. This demonstrates the coexistence of two phases during the decompression. The process was reversible and, under atmospheric pressure, all parameters returned to their initial value.

**Fig. 4:**
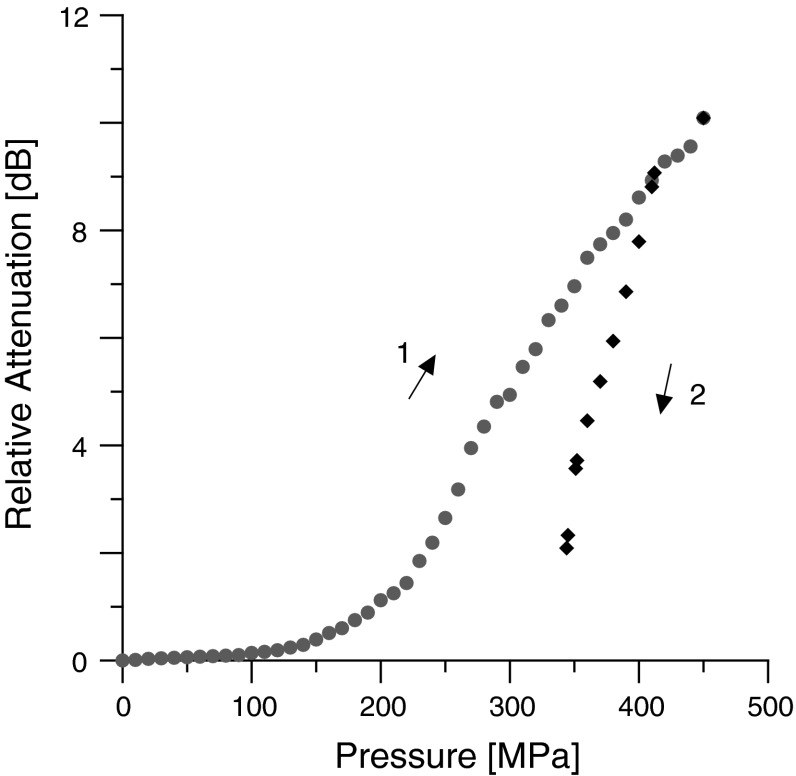
Pressure dependence of the relative sound attenuation in olive oil. *T* = 293 K, *f* = 5 MHz. *Arrows* indicate the direction of pressure changes. *1* low-pressure phase, *2* phase transition

The pressure dependence of the relative sound attenuation in olive oil is presented in Fig. 4. As can be seen in Fig. 4, at a pressure of 450 MPa, attenuation shows discontinuity. This can be evidence of the existence of a phase transition.

On the basis of the above measurements the density, the specific volume, and adiabatic compressibility (from the sound velocity) were evaluated.

**Fig. 5:**
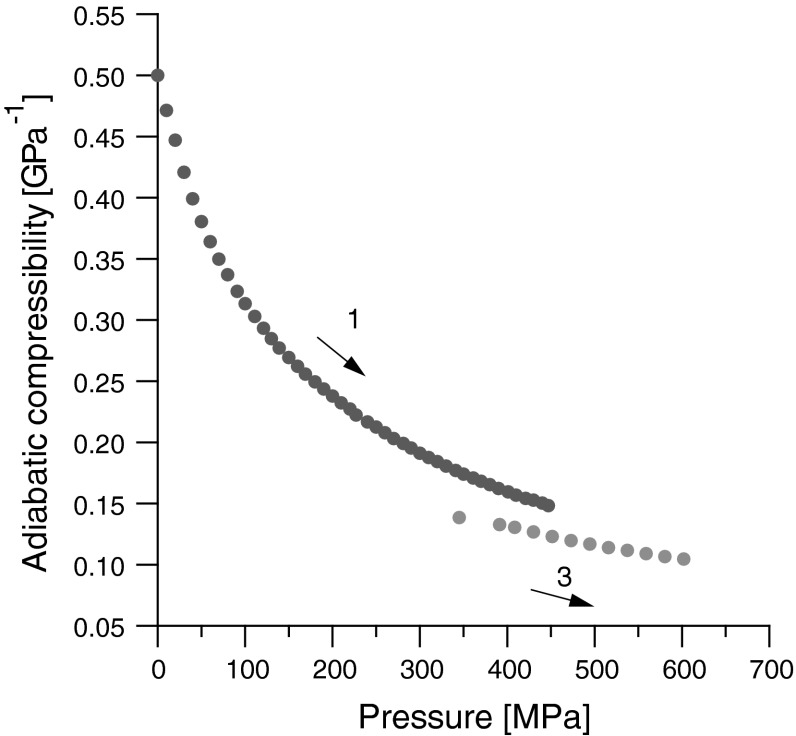
Adiabatic compressibility of olive oil versus pressure, *T* = 293 K. The direction of the pressure changes is similar to that in Fig. 1. *1* low-pressure phase, *3* high-pressure phase

Dependence of adiabatic compressibility on pressure is presented in Fig. 5. As one can notice the compressibility after the phase transition is much smaller and less dependent on pressure than below the phase transition.

**Fig. 6:**
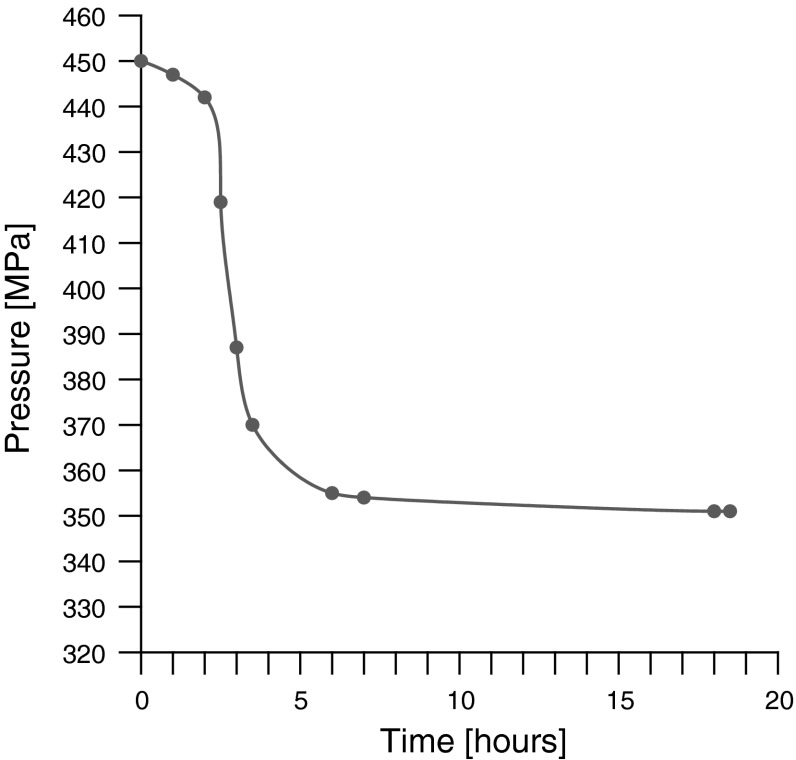
Kinetics of phase transition in olive oil (*T* = 293 K)

The kinetics of the phase transition is presented in Fig. 6. The phase transition in triolein which is the main component of vegetable oils takes a short time (about 40 min) [6]. Therefore, this phase transformation was discovered very quickly. On the other hand, the phase transition in other types of vegetable oils (e.g. castor oil [3]) takes a much longer time (e.g., even a few days). Therefore, the discovery of phase transitions in these oils has only been made relatively recently. Olive oil is also included in this type of oils. Phase transformation in olive oil takes approximately 6 h.

## Conclusions

The following conclusions arise from the conducted investigations:

1. Dependencies of sound velocity, ultrasonic wave attenuation, volume, and adiabatic compressibility on pressure show discontinuities. This proves the existence of a first order phase transition in olive oil (liquid to solid-like phase transition).
2. The biggest relative changes were observed for the dependence of ultrasonic wave attenuation on pressure. Rapid and large changes in attenuation at a fixed position of the piston testify to the phase transition occurring in olive oil.
3. Values of the pressure during the phase transition are very similar to that of triolein since in both cases, triacylglycerols of oleic acid are the main constituents of triolein and olive oil.
4. The induction time of the phase transition in olive oil is greater than that in the case of triolein because of the different chemical composition of these oils.
5. The phase transition in the examined olive oil at 20 °C occurred at a pressure of 450 MPa. In our present and previous [6, 19, 29] investigations, it was shown that in triacylglycerol at 20 °C, the phase transition occurred in the same pressure range [6], in diacylglycerol - at about 210 MPa [8]. The transformation time for olive oil was about 6 h, for triacylglycerols, it was about 40 min. [6], and for diacylglycerol it was approximately 20 min [8].

The investigation of phase transitions is very important in high-pressure food processing and conservation. Phase transformations can modify the molecular structure significantly and consequently, affect the texture and sensory characteristics of food products [11]. Therefore measuring the speed of sound in the liquid at high pressure enables one to control the quality of food products subjected to high-pressure technological processes, and to understand the nature of the physicochemical behavior of oils.

The only article on high-pressure phase transitions in olive oil known to the authors was an article [18]. However, in the article [18], the composition of the investigated oil is not given. In contrast to [18], the authors of the present paper did not observe the presence of the phase transition at a pressure value of 124 MPa. In our experiments, the phase transition in olive oil (for a temperature of 20 °C) started at higher pressure values, i.e., at about 450 MPa. Although the authors of the work [18] investigated pressures as high as 1,167 MPa, they only included points up to 356 MPa in their analysis. In this range of pressure, a similar trend in velocity with pressure was observed as in the present paper. Also in the work [30], the phase transition was not observed in the olive oil in a pressure range from atmospheric pressure up to 150 MPa. In this work the relationship of viscosity with pressure for olive oil up to 150 MPa was studied. The authors did not observe any discontinuities in viscosity values. Such discontinuities always accompany the phase transitions in oils. The authors of [30] could not measure the viscosity at higher pressures due to inherent limitations of the applied measuring method. They used a falling sinker viscometer. The discrepancy with the results of [18] may be due to a different composition of the oil used in the study by the authors of [18] and the authors of the present work.

Quantitative comparison of results is valid only for liquids with known compositions. Olive oils may differ in composition (content of fatty acids, triacylglycerols, etc.), and thus in their properties [28]. A similar conclusion is included in the work [13].

In our work we have given the composition of the investigated oil determined by gas chromatography. This will allow the comparison of our results with those obtained for oils of similar composition. To our best knowledge, such results have not yet been published.
